# A Preliminary Pilot Randomized Crossover Study of Uzara (*Xysmalobium undulatum*) versus Ibuprofen in the Treatment of Primary Dysmenorrhea

**DOI:** 10.1371/journal.pone.0104473

**Published:** 2014-08-13

**Authors:** Karim H. I. Abd-El-Maeboud, Mohamed A. M. F. Kortam, Mohamed S. Ali, Mostafa I. Ibrahim, Radwa M. M. Z. Mohamed

**Affiliations:** Department of Obstetrics and Gynecology, Faculty of Medicine, Ain Shams University, Cairo, Egypt; Karolinska Institutet, Sweden

## Abstract

**Objective:**

Preliminary evaluation of efficacy and safety of uzara use in treatment of moderate and severe primary dysmenorrhea in comparison to ibuprofen.

**Materials and Methods:**

This randomized, comparative two way cross-over study comprised 60 single female students at Faculty of Medicine, Ain Shams University, Egypt, aged 19–28 years with moderate (n = 46) or severe (n = 14) primary dysmenorrhea. Participants were randomized to take either uzara (80 mg/8 hours for two doses, then 40 mg/8 hours) then ibuprofen (400 mg/6 hours) in two subsequent cycles or vice versa. The pain intensity, using VAS, was recorded immediately before taking the medication (0 hour) and after 4, 12, 24, 48–60, 96–120 hours. Main outcome measures included effectiveness of pain relief defined as drop of VAS to 3 or less, patient's global evaluation of the drug, absence from school, the use of a rescue medication, and, in those who continued the treatment, the pain intensity difference (PID) at different points after start of medication and its sum (SPID).

**Results:**

Uzara was comparably effective to ibuprofen (78.3% vs. 86.7% of cycles; respectively), with comparable rates of effectiveness on global evaluation (being around 50% for either drug), and rates of school absences (11.7% vs. 13.3%; respectively). The need for rescue medication was different (18.3% and 10%; respectively), albeit with no statistical significance. The means of PID at different time points and SPID were comparable, with significantly lower average mean of VAS scores compared to that felt with no medication (1.6 vs. 6.8, p<0.001). Side effects were less with uzara than ibuprofen (0% vs. 8.3%, p<0.05).

**Conclusions:**

Uzara might be as effective as ibuprofen in management of primary dysmenorrhea but with less side effects. These findings need to be confirmed by a properly designed trial with a larger sample size.

**Trial Registration:**

Current Controlled Trials ISRCTN25618258

## Introduction

Primary dysmenorrhea is a common gynecological problem [1]–[3]. Nonsteroidal anti-inflammatory drugs (NSAIDs) are well established as first-line therapy. Common side effects are nausea, vomiting, dyspepsia and diarrhea; with less common serious ones [4]. They act by suppressing the production of endometrial prostaglandins [2]. They are significantly more effective in relieving pain when compared to placebo or paracetamol. However, they have an overall treatment failure rate of approximately 25%. Regarding effectiveness and safety, evidence is still lacking to determine the best individual NSAID in treatment of dysmenorrhea [2], [5]. The combined oral contraceptives pills (COCPs), which act by decreasing menstrual fluid volume and prostaglandin production have been advocated to treat primary dysmenorrhea, however there is insufficient evidence for pain relief with their use [6]. Other alternative therapeutic modalities include acupuncture [7], transcutaneous electrical nerve stimulation (TENS) [8], laparoscopic presacral neurectomy [9], spinal manipulation [10], behavioral interventions [11], Zingiber officinale R. rhizomes (ginger) [12], and herbal and dietary therapies (including magnesium, vitamin B6, vitamin B1, vitamin E, omega-3 fatty acids, and Japanese herbal combination) [13].

Uzarae radix. is a herbal remedy extracted from the uzara root, which constitutes of the dried underground parts of the 2–3 year-old plants Xysmalobium undulatum, This important medicinal plant has been historically used by the native inhabitants of South Africa to treat digestive complaints. It is approved by Commission E to treat acute nonspecific diarrhea [14]. Although the inhibition of active chloride secretion has been recently suggested to play a role [15], the anti-diarrheal action of uzara root extracts is thought to be mainly through inhibiting the motility of visceral smooth muscle. The action is qualitatively similar to that of papaverine [16]. Uzara root extracts inhibit motility in the small intestine and urogenital tract with traditional uses including afterbirth cramps and dysmenorrhea [17]. Strangely enough, there are no published studies evaluating its use as a treatment option for menstrual cramps although its use has been suggested since 1913 [18]. Also, no health hazards or side effects are known [19] and recent studies have failed to find significant cardiovascular pharmacodynamic effects with its oral administration [20]. Uzara contains glycosides with cardenolide structure (uzarin and xysmalorin) which have been reported to cross-react with the conventional digitalis assays [20], [21], thus not to be administered concomitantly with other cardioactive glycosides [14], [21].

The aim of this study is to evaluate the efficacy, safety and tolerability of uzara in comparison to ibuprofen, a NSAID, in the treatment of primary dysmenorrhea.

## Materials and Methods

This was a pilot phase III randomized comparative, two way cross-over assignment, safety and efficacy study, aiming at preliminary evaluation of uzara versus ibuprofen in the treatment of primary dysmenorrhea. The protocol for this trial and supporting CONSORT checklist are available as supporting information; see Protocol S1 and Checklist S1.The study protocol approval was obtained on 29^th^ August 2010 (FMASU 773/2010) from the Ethics and Research Committee of the Faculty of Medicine, Ain Shams University 00006444. The authors confirm that all their ongoing and related trails for uzara in their institute are registered. The participants were 60 women with moderate or severe primary dysmenorrhea, recruited from female students attending clinical classes in medicine (Faculty of Medicine) or nursing (High Institute of Nursing). The inclusion criteria were: age between 18 and 28 years, regular cycles (21–35 days) with duration of 3 to 7 days, a history of at least 6 consecutive months of moderate to severe primary dysmenorrhea as determined by the verbal rating scale (VRS, a 4-point self-rated verbal score: 0, none; 1, mild; 2, moderate; and 3, severe menstrual pain) [22], with the pain lasting for at least 2 days and who required analgesia in each of the last 3 consecutive cycles, preceding participation in the study. The exclusion criteria were planning to get married during the study period; known or suspected secondary dysmenorrhea and other causes of chronic pelvic pain (previous major abdominal or pelvic surgery, endometriosis, pelvic inflammatory disease, ovarian cysts, pathological vaginal secretion, chronic abdominal pain, inflammatory bowel disease, irritable bowel syndrome); hormonal therapy during the last 6 months or planning to take hormonal therapy during the study period; regular intake of pain medications (including NSAIDs) for other reason than dysmenorrhea, digoxin, antidepressants, tranquilizers, hypnotics, sedatives, or sex hormones; any concomitant disease or condition that might require any intake of analgesic medication; history of significant chronic constipation or recurrent colitis; evidence of clinically relevant serious condition whether gynecological, cardiovascular, hematological, hepatic, gastrointestinal, renal, pulmonary endocrinological, autoimmune diseases, neurologic or psychiatric disease, based on a clinical assessment and routine laboratory investigations; unwillingness to comply with the protocol, or participation in another clinical trial in the 3 months prior to the start of this study.

Each participant completed a case record form, including demographic details and past medical and surgical history. A transabdominal ultrasonographic scanning (TAS) was performed in all participants to exclude any pelvic pathology. Severity of dysmenorrhea in all participants was initially assessed by two validated pain scales: visual analogue scale (VAS), verbal rating scale (VRS) [23]. VAS is a one-dimensional linear scale of 0–10 cm (0 cm representing no pain; 10 cm, severe pain) with values greater than 5 defined as moderate or severe. Marked limitation of daily activities evidenced by absence from school was recorded.

The objective of the study was explained to eligible participants, who all signed a written informed consent. The randomization sequence was computer generated and kept concealed by the first author (KA) who played no role in patients' recruitment. Other co-authors -indulged in patients' recruitment and consenting to the study- were continuously updated regarding number of participants with assigned sequence. They collaborated together to allocate the next available number to each participant in order of her enrollment in the study. Subsequently, the first author (KA) was contacted and asked to release the sequence (order of drug intake, uzara/ibuprofen or vice versa).

Uzara is a coated tablet, contained purely vegetable active substance 40 mg dry extract of roots of uzara plant, *Xysmalobium undulatum* (4–6∶1) [extracting medium: methanol 60% (V/V)] and glucose, lactose, sucrose, and fortifying wheat (Uzara 40 mg, Registration owner: STADA GmbH, Bad Vilbel, Manufacturer: HEMOPHARM GmbH, Germany). The control drug was ibuprofen 400 mg sugar coated tablets (Brufen 400 mg, Kahira Pharm. & Chem. Ind. Co., Cairo, Egypt; under license from Abbott Laboratories). Treatment was commenced with the onset of menstruation or when pain is felt to be impending (up to two days before the expected start of menstruation) and stopped when there was mild or no pain (VAS score 3 or less) 6–8 hours from the last test tablet or after 5 days treatment duration. Both drugs were taken orally; uzara two tablets every 8 hours for 2 doses then one tablet every 8 hours (maximum daily dose 200 mg), Ibuprofen one tablet every 6 hour (maximum daily dose 1600 mg). Each of the two drugs, ibuprofen and uzara, has a short serum half-life, being 1.8–2 hours [24] versus 8.87 ± 2.20 hours [25]; respectively. It is reported that the duration of washout time should be approximately 10× the plasma apparent terminal elimination half-life to provide for 99.9% of the administered dose to be eliminated from the body [26]. So, the incorporation of a relatively long washout period (3 weeks between treatment periods, i.e. two successive menses) in the cross over design of the present study was assumed to diminish safely the impact of carryover effects and eliminate the need for testing for carry over effects. In both treatment cycles, participants recorded the pain intensity they experienced, using visual analogue scale (VAS) at 6 time-points: immediately before starting the tested medication (0 hour) and after 4, 12, 24, 48–60 and 96–120 hours. The occurrence of school absence as a result of confirmed marked limitation of physical activity in relation to dysmenorrhea was recorded. The use of other analgesics was allowed for those receiving little or no pain relief i.e., rescues medication. This was permitted 1 hour after the intake of the tested drug. The type, dose, frequency of administration and the response using VAS were recorded after the intake of the rescue drug, if needed. Adverse reactions and tolerability to all medications were recorded. After the end of the each treatment cycle, participants' global evaluation of the study medication was recorded as effective or non-effective.

In the present study, the primary outcomes measures –all related to analgesic effect- were: a drop in VAS score to ≤3 at point B (4 hours after receiving the medication), participant's global evaluation of the drug, absence from school, the use of a rescue medication, and in those who continued the treatment, the pain intensity difference (PID) at certain points after start of medication and its sum (SPID). Secondary outcome measures included adverse reactions and drug tolerability recorded at the end of each cycle.

Being a preliminary pilot study to assess the use of uzara in dysmenorrhea compared to the standard therapy (ibuprofen) in knowledgeable medical students, who might be skeptic about its effectiveness, we opted to include a sample size (60 subjects) larger than usual to avoid a false negative result which would preclude the opportunity to further examination of uzara in this respect. All analyses were performed with SPSS 17 (SPSS, Inc., Chicago, IL, USA). The Student t tests (for independent and dependent parameters) were used for continuous variables whereas Fisher's exact test was used for categorical variables. For all analyses, a two-tailed *p* value of less than 0.05 was considered statistically significant. Intention-to-treat (ITT) analysis was performed for all outcomes except for pain intensity difference (PID) over time. The latter was analyzed in those receiving study medications (uzara versus ibuprofen) as “modified intention-to-treat” or “per protocol”; a non-randomized, observational comparison. For those receiving rescue medications, as pre-specified in study protocol, the response using VAS scoring was recorded and analyzed separately to avoid confounding the analgesic effect of study medications. Risk analysis was done to estimate the risk of untoward outcomes in either study group. The relative risk (RR) was then calculated as the risk in the uzara group relative to the ibuprofen group. Absolute risk reduction (ARR) was calculated by subtraction of the risk in the ibuprofen group from that in the uzara group. The number needed to treat (NNT) was then calculated as the reciprocal of the ARR. All risk estimates were presented along with their 95% C.I. (confidence interval).

## Results

**Figure 1:**
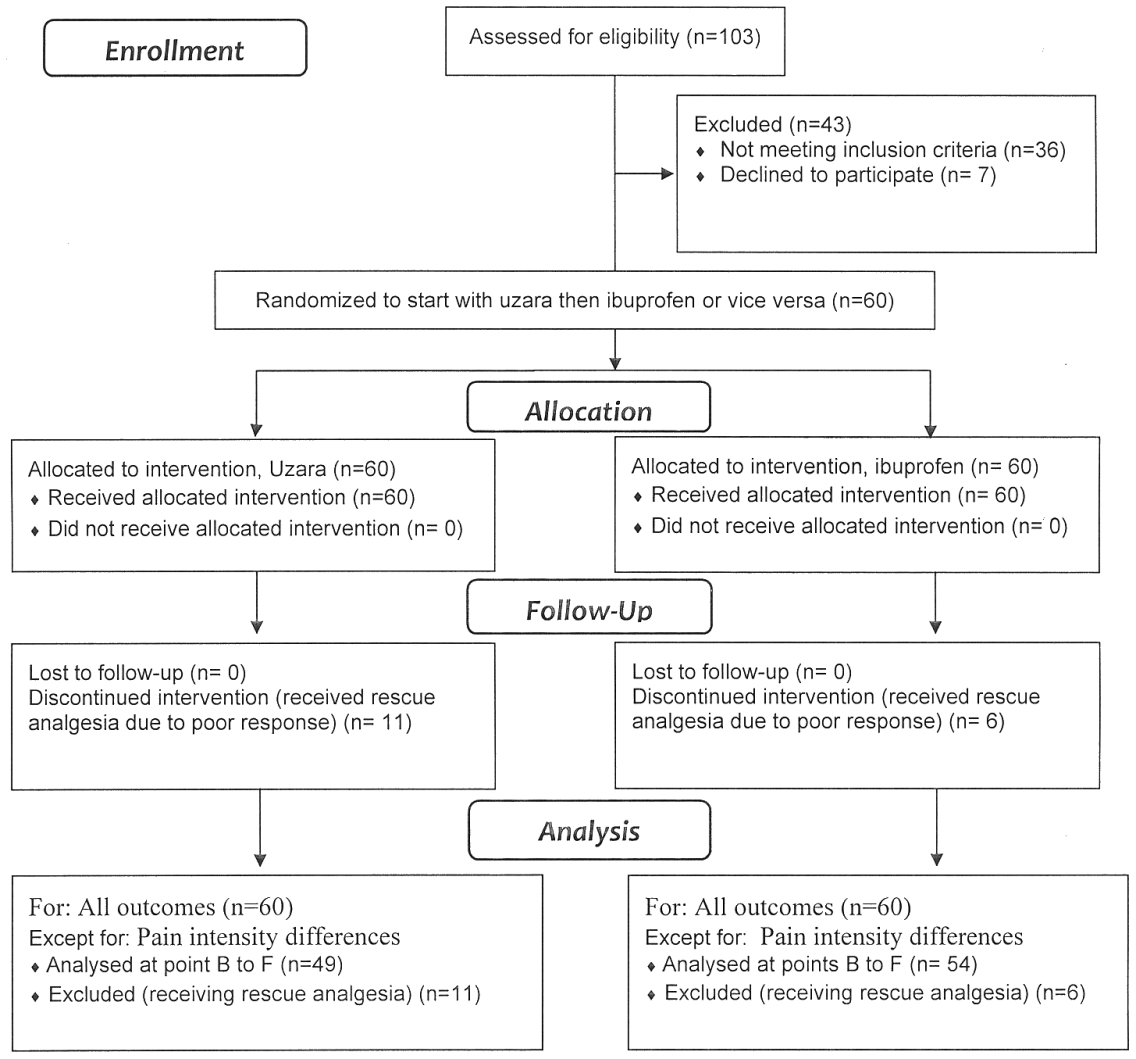
Flow-Chart of study participants.

To save time as the researchers were late in registering the study internationally, the study was announced during lectures so that those students suffering from dysmenorrhea and willing to volunteer in a trial of a new therapeutic line were invited to register themselves in a preliminary list for interview. This lasted till January 31^st^ 2011when the trial was registered (Trial registration number: ISRCTN25618258; Registry website: http://www.controlled-trials.com). Following this, the first 103 subjects, in order, were interviewed with full explanation of the study protocol and assessment for eligibility. Patients' enrollment (60 subjects) followed with signing a well-informed consent, use of medications, and follow up till March 28, 2012 when the trial ended (Figure 1). Initial assessment, using VRS scoring, revealed that dysmenorrhea was moderate in 46 patients and severe in 14 patients, all virgin with absence of prior sexual intercourse. The general characteristics of the participants are shown in Table 1. Medical advice was sought in 16.7% (10/60); with an analgesic being prescribed to the participants by a lay relative in 85% (51/60) of cases. Family history of primary dysmenorrhea - in mother, a sister, or both – was positive in 50% of participants (30/60).

**Table 1:** Participants' Characteristics (n = 60).*

Characteristic	Median/No.	IQR/Percent
**Age** (years)	23	20.5–24
**BMI**	22.5	21.1–25.6
**Age at menarche** (years)	12	11–13
**Cycle** (days)	28	27–28
**Flow** (days)	6	5–7
**Flow:** Average	52	86.7
Heavy	8	13.3
**Age at Onset of Dysmenorrhea** (Years)	13	12–14
**Duration of Dysmenorrhea** (days):		
One	14	23.3
Two	32	53.3
Three	11	18.3
Four	3	5
**Onset of Dysmenorrhea in relation to Onset of Menses:**		
−2 days	11	18.3
−1 day	16	26.7
Same day	26	43.3
+1 day	6	10
+2 days	1	1.7
**Peak of Dysmenorrhea in relation to Onset of Menses:**		
−2 days	3	5
−1 day	9	15
Same day	32	53.3
+1 day	11	18.3
+2 days	5	8.3
**Peak of Dysmenorrhea in relation to Flow of Menses:**		
Mild flow	21	35
Moderate flow	22	36.7
Heavy flow	17	28.3
**Absence from Work during Menses:**		
None	17	28.3
Occasional	24	40
Alternate menses	8	13.3
Every menses	11	18.3

* Results are median/number (No) and interquartile range (IQR)/percentage (percent)

**Figure 2:**
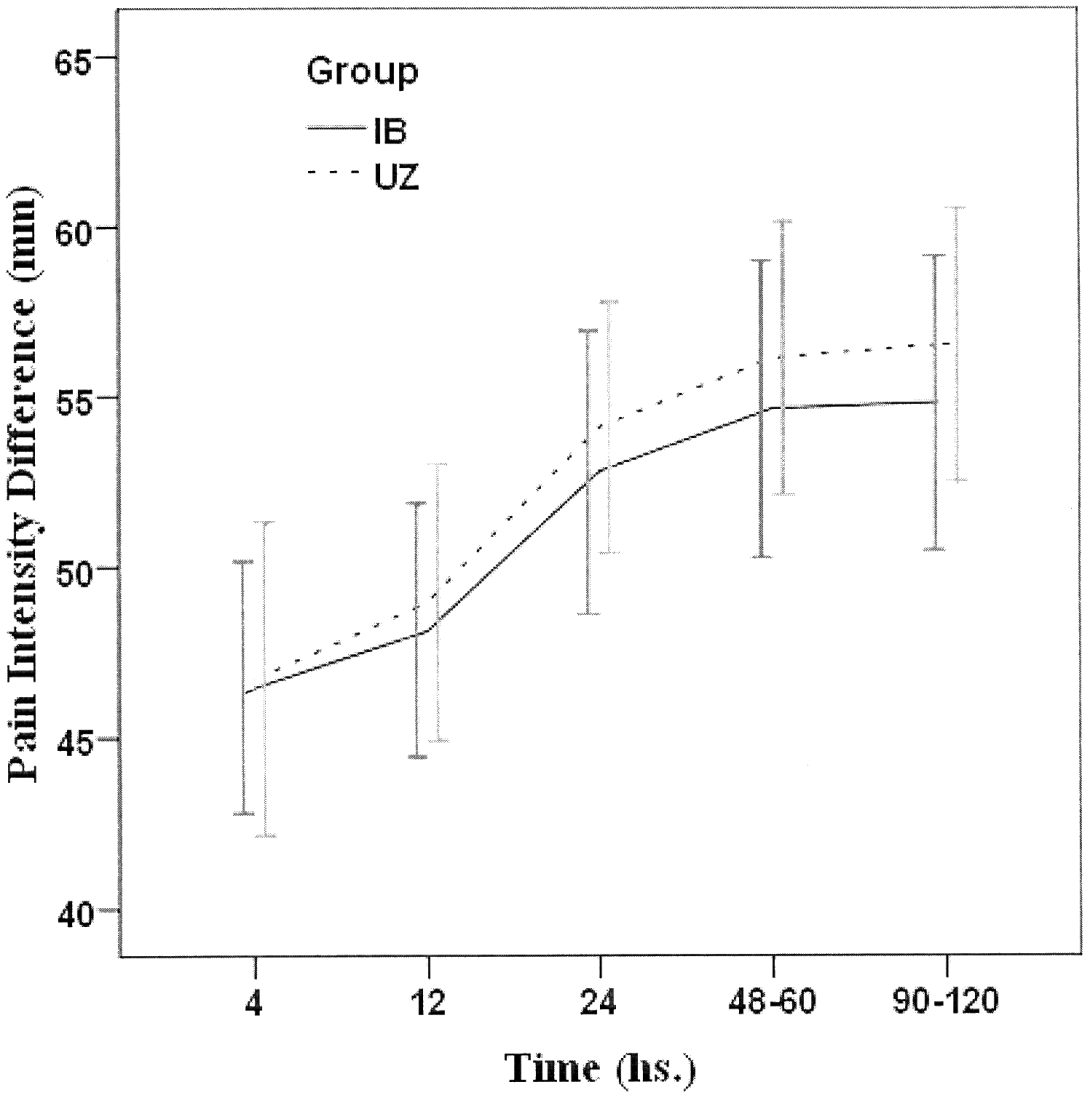
Pain intensity difference (PID) – mean scores of visual analog scale (error bars: 95% C.I., confidence interval) based on drug start at each cycle with Uzara (UZ) or Ibuprofen (IB) (Cycles: UZ, *N = 49*; IB, *N = 54*), excluding 17 cycles where rescue analgesia was used.

Thirty participants started with uzara, while the other 30 started with ibuprofen. There was no significant difference between uzara and ibuprofen cycles regarding the number of doses taken by the participants (2.12 ± 0.97 vs. 2.28 ± 0.76, p = 0.371; with median being 2 and range 1–4 vs. 1–6; respectively). The effectiveness of uzara and ibuprofen were comparable (Table 2) in terms of the reduction of VAS score to 3 or less (78.3% vs. 86.7%; respectively, p = 0.337) and participant's global evaluation (48.3% vs. 51.7%; respectively, p = 0.855). Satisfactory relief of pain, as indicated by the absent need of rescue drug, was lower with uzara compared to Ibuprofen (81.7% vs. 90%) groups, albeit with no statistical significance (p = 0.295). Of note, 2 (3.3%) participants with severe dysmenorrhea received a rescue drug with both tested medications, i.e. both medications failed to relief their pain. Discordant response with only one drug being effective and reported failure with the other was also noticed; uzara alone was effective in 2 participants, while ibuprofen only was effective in 9 participants. School absence rates were comparable; being 11.7% (7/60) for uzara and 13.3% (8/60) for ibuprofen, (p>0.05); both being lower than the participants' historical school absence rate (19/60 or 31.7%; p<0.01). In those who continued the study medications, the means of pain intensity difference (PID) at different time points after their start and its sum (SPID) were comparable (Figure 2, and Table 3). The mean post-treatment VAS scores in both uzara and ibuprofen cycles were comparable (1.63±0.86 vs. 1.61±0.83; respectively), being both significantly lower than the initial (pre-treatment) VAS (6.77±1.57; p<0.001). In those using rescue medications, the average VAS scores after drug use were 2.7±2.7 and 3±0.8; respectively (p>0.05).

**Table 2:** Risk analysis for the main outcome measures (Direction: Uzara minus Ibuprofen).

	Uzara group (n = 60)				Ibuprofen group (n = 60)									
Outcome	Favorable outcome	Unfavorable outcome	AR	95% CI	Favorable outcome	Unfavorable outcome	AR	95% CI	RR	95% CI	ARR	95% CI	NNT	95% CI
**VAS**	47(78.3%)	13 (21.7%)	0.22	0.11 to 0.32	52 (86.7%)	8 (13.3%)	0.13	0.05 to 0.22	1.63	0.73 to 3.63	−0.08	−0.22 to 0.05	−12.0	−4.58 to 19.30
**Global outcome**	29 (48.3%)	31(51.7%)	0.52	0.39 to 0.64	31(51.7%)	29 (48.3%)	0.48	0.36 to 0.61	1.07	0.75 to 1.53	−0.03	−0.21 to 0.15	−30.0	−4.71 to 6.87
**School absenteeism**	53 (88.3%)	7 (11.7%)	0.12	0.04 to 0.20	52 (86.7%)	8(13.3)	0.13	0.05 to 0.22	0.88	0.34 to 2.26	0.02	−0.10 to 0.13	60.0	−9.84 to 7.41
**Rescue analgesic use**	49 (81.7%)	11 (18.3%)	0.18	0.09 to 0.28	54 (90%)	6 (10%)	0.10	0.02 to 0.18	1.83	0.72 to 4.64	−0.08	−0.21 to 0.04	−12.0	−4.83 to 24.66

AR, absolute risk; ARR, absolute risk reduction; CI, confidence interval; NNT, number needed to treat; RR, relative risk.

**Table 3:** Effectiveness based on pain intensity difference (PID) at different time points after drug start and their sum (SPID) with Uzara (UZ) and Ibuprofen (IB) (Cycles: UZ, *N = 49*; IB, *N = 54*), with exclusion of 17cycles where rescue analgesia was used.

Effectiveness measure (Visual analogue sscale)	Uzara (Mean ± S.E.M.)	Ibuprofen (Mean ± S.E.M.)	P	Mean Difference (95% C.I.)
**Basal VAS**	67.35 ± 2.12	66.30 ± 1.93	0.714	1.051 (−4.622 to 6.723)
**Pain Intensity Difference (PID) by hour after drug intake:**				
**4 hs.**	46.73 ± 2.29	46.48 ± 1.83	0.931	0.253 (−5.511 to 6.018)
**12 hs.**	48.98 ± 2.03	48.15 ± 1.85	0.762	0.831 (−4.602 to 6.265)
**24 hs.**	54.08 ± 1.84	52.78 ± 2.07	0.642	1.304 (−4.240 to 6.848)
**48–60 hs.**	56.12 ± 2.02	54.63 ± 2.18	0.618	1.493 (−4.427 to 7.413)
**90–120 hs.**	56.53 ± 2.01	54.81 ± 2.16	0.565	1.716 (−4.172 to 7.604)
**Sum (SPID)**	262.45 ± 9.19	256.85 ± 9.6	0.676	5.597 (−20.887 to 32.081)

CI, confidence interval; P, probability; S.E.M., standard error of mean.

All participants tolerated uzara well with 0% (0/60) side effects compared to 8.3% (5/60) with ibuprofen (p<0.05). These were all gastro-intestinal side effects including nausea (two participants), vomiting (one participant), stomach cramps alone (one participant) or with nausea (one participant).

## Discussion

This study has assessed the efficacy, safety and tolerability of uzara in comparison to ibuprofen, a NSAID, in the treatment of primary dysmenorrhea. Our analyses showed that the effectiveness of uzara was comparable to that of ibuprofen, a standard first line therapy. The failure rates of uzara (21.7%) and Ibuprofen (13.3%) were comparable to other reported NSAIDs [2], [5]. Two participants failed to respond to both drugs, while discordant response with only one drug being effective was also noticed in 11 participants. All participants tolerated uzara well with no reported side effects compared to 8.3% in Ibuprofen cycles.

A point of strength of our study is the prospective randomized design with crossover assignment of medication to allow each participant to act as her own control. Also, to provide clinically meaningful results, two measurement of severity of dysmenorrhea, visual analogue scale (VAS) and verbal rating scale (VRS), were used [22]. Although, some investigators considered a reduction of 3 points of pain severity in the VAS as an outcome to define successful treatment [23], a reduction of VAS score to 3 or less was chosen to indicate effectiveness in the present study as this would indicate a mild degree of pain likely to be acceptable to the woman. Retrospective participant's global evaluation of each medication showed also comparable efficacy; albeit with lower figures which might be explained by subject's expectation to have complete analgesia. One limitation is failure to double blind this study, due to lack of technical facilities to manufacture ibuprofen tablet identical to that of uzara. Non-blinding of the participants might have being disadvantageous to uzara, being acknowledged by them as a test medication with potential loss of trust and lowered expectations regarding efficacy. This could be a source of bias against uzara with apparently less effectiveness (78.3% vs. 86.7% of cycles of ibuprofen; respectively), and higher need for rescue drug usage (18.3% and 10%; respectively), though being statistically insignificant. Other limitations are the apparently lower number of study participants and belonging to a special cultural sector with medical background. Since all study participants were virgin with absence of prior sexual intercourse, an assumption based on strict religious banning of pre-marital sex in our society, our results can be safely generalized to unmarried or abstinent young females. In fact, one might assume that this would not interfere with overall generalizability due to elimination of other confounding factors, such as IUD or pelvic infection, which can interfere with dysmenorrhea.

We hypothesized that uzara is effective in treating primary dysmenorrhea. Catecholamines can theoretically cause either excitation or inhibition of the uterus depending on whether alpha or beta adrenergic receptors predominate. The adrenergic responsiveness of the myometrium appears to be regulated by steroid hormones [27]. It is suggested that stress-associated enhancement of sympathetic activity leads to exacerbation of uterine contraction and increased menstrual pain. This activity might be decreased via exercise with subsequent symptomatic relief [28]. The mechanism of analgesic effect of uzara is apparently through inhibiting the motility of visceral smooth muscle of the uterus, probably via the local stimulation of sympathetic nerves with an inhibitory effect on smooth muscles; an action similar to that suggested for its anti-diarrheal action [16]. This is further supported by earlier use of beta2-adrenoceptor agonists in the treatment of women with primary dysmenorrhea. Despite the failure of hydroxyphenyl-orciprenalin [29], and isoxsuprine [30], to show any benefit in previous studies, another one reported pain relief with a combination of isoxsuprine, acetaminophen and caffeine [31]. Moreover, two studies reported pain relief in severe primary dysmenorrhea with terbutaline administered via spray inhalations [32] or intravenous injection, along with inhibition of the myometrial activity and increased uterine blood flow [33]. A recent review on the use beta2-adrenoceptor agonists for dysmenorrhea found that adverse effects with all these medications were reported in up to a quarter of the total number of participants and they included nausea, vomiting, dizziness, tremors and palpitations. Although this mechanism for relieving dysmenorrhea fell into disrepute for over 30 years due to high incidence of adverse effects in these few, relatively small-sized and inadequate quality studies [34], this does not preclude the assertion that uterine sympathetic supply plays a role in the pathogenesis of primary dysmenorrhea. The response to uzara in the present study supports this view. Therefore, uzara might benefit women by relieving dysmenorrhea via this mechanism of action, but without significant risk of side effects, which preclude the use of beta2-adrenoceptor agonists as an acceptable clinical treatment option.

This study suggests that uzara might be as effective as ibuprofen in treatment of primary dysmenorrhea. Considering its better tolerability, shorter contraindication list and lower incidence of side effects, uzara might be considered as an alternative agent to NSAIDs in women who have failed response, contraindications or side effects. With suggested different mechanism of action of uzara, one might wonder whether combining it with NSAIDs would have a good additive effect evidenced by a better success rate and the need of lower doses of NSAIDs with lower adverse effects. Therefore, further large-scale studies are warranted to confirm and explore uzara's role in the management of primary dysmenorrhea.

## Supporting Information

Checklist S1 **CONSORT Checklist.**
(DOC)

Protocol S1 **Trial Protocol.**
(DOC)
