# Supplementary material for: A Preliminary Pilot Randomized Crossover Study of Uzara (Xysmalobium undulatum) versus Ibuprofen in the Treatment of Primary Dysmenorrhea
Source: PLoS One. 2014 Aug 13;9(8):e104473. doi: 10.1371/journal.pone.0104473 (PMC4131898; doi:10.1371/journal.pone.0104473)
Supplement: Protocol S1 — Trial Protocol. (DOC) [file pone.0104473.s002.doc]

**Uzara (Xysmalobium undulatum) versus Ibuprofen in the treatment of primary dysmenorrhea among Egyptian Medical University Students:**

**A randomized, crossover study**

Protocol for a Clinical Trial

**Prof. Karim Hassnein Ismail Abd-El-Maeboud**

Professor of Obstetrics and Gynecology,

Ain Shams University

**Prof. Mohamed Ashraf Mohamed Farouk Kortam**

Professor of Obstetrics and Gynecology,

Ain Shams University

**Dr. Mohamed Sayed Ali**

Assistant Professor of Obstetrics and Gynecology,

Ain Shams University

**Dr. Moustafa Ibrahim Ibrahim**

Assistant Professor of Obstetrics and Gynecology

Ain Shams University

**Radwa Mansour Mohamed Zaki Mohamed**

MSc. OB/GYN

Assistant lecturer in OB/GYN Department

Ain Shams University Maternity Hospital

Department of Obstetrics and Gynecology,

Faculty of Medicine

Ain Shams University

2010

**INTRODUCTION**

Dysmenorrhea is one of the most frequent gynecologic disorders affecting more than half of menstruating women. Most adolescents experience dysmenorrhea in the first few years after the menarche **(Ozgoli et al, 2009).** It is defined as a pelvic pain directly related to menstruation. It is associated with symptoms like headache back ache, nausea, vomiting and diarrhea. It is classified into two categories; primary when pelvic examination and ovulatory function are normal and secondary; when there is an identifiable gynecological pathology **(Durain, 2004).** Prevalence of primary dysmenorrhea is reported in many studies to vary between 50 and 90 %. **(Davis and Westhoff, 2001).** In an epidemiological study that entailed 664 adolescent female students in secondary schools in urban and rural areas in Mansoura in Egypt, it is found that about 75% of the students have dysmenorrhea, being mild in 55.3%, moderate in 30%, and severe in 14.7% **(El-Gilany et al, 2005).**

Primary dysmenorrhea characteristically begins when adolescents attain their ovulatory cycles; generally within the first year after menarche. **(Durain, 2004).** The pain is believed to result from excessive prostaglandin release, particularly PGF2 α**. ( Dawood, 2006; Harel, 2006).** As progesterone concentration falls before menstruation, arachidonic acid is released from the endometrial cell membranes and a cascade of prostaglandin synthesis is initiated in the uterus. (**Harel, 2006).** In comparison to women with eumenorrhea, women with dysmenorrhea have higher concentrations of PGF2 α in their menstrual fluid**.** **(Nigam, 1991; Biegelmayer et al, 1995).** PGF2 α causes vasoconstriction of uterine blood vessels (uterine ischemia) and increases uterine smooth muscle contraction, **( Funk, 2001; Harel ,2002)** and it is the contraction of the ischemic uterus that is likely the cause of dysmenorrhea. **(Dawood, 2006).**

Being a gynecological problem, primary dysmenorrhea is an important health problem concerning public health, occupational health and family practice, since it affects both the quality of life and the national economy due to short-term school absenteeism and loss of labor **(Fedele et al, 1990; Marchini et al, 1995).** Unfortunately, both the prevalence of primary dysmenorrhea and the manner in which females attempt to solve this problem are unknown in most of the developing countries. **(Fedele et al, 1990; Marchini et al, 1995).**

Non-steroidal anti-inflammatory drugs (NSAIDs), which decrease the formation of PGF2 α, are effective in alleviating dysmenorrhea **(Banikarim et al, 1995;** **Houston et al, 2006).** Non-steroidal anti-inflammatory drugs (NSAIDs) are widely used as first-linetherapy in women with primary dysmenorrhea. Evidence based data support theefficacy of ibuprofen, naproxen, mefenamic acid, and acetyl salicylic acid **(Dawood, 2006).** These drugs, however, have side effects, of which gastrointestinal disorders suchas nausea, dyspepsia, and vomiting are the most common **(Burke et al, 2006).** Some patients with primary dysmenorrhea do not respond to treatment with NSAIDs. In addition, some women have contraindications to these medications. Consequently, researchers have investigated numerous alternative/complementary treatments such as herbal and dietary therapies**, (Proctor et Murphy, 2001),** behavioral interventions, **(Proctor et al, 2007),** acupuncture**, (Taylor et al, 2002),** and aromatherapy **(Han et al, 2006).**

*Xysmalobium undulatum* (L.) –Uzara, is an important medicinal plant (drug –extract of the roots of the South-African Uzara plant) which has a long history of traditional and commercial use, not only in South Africa (where it has been cultivated since1904), but also in Germany, **(Dennehy, 2006).** Uzara root consists of the dried, underground parts of 2- to 3-year-old plants of Xysmalobium undulatum. Uzara inhibits intestinal motility. In high dosages, it has a digitalis-like effect on the heart. It is approved by Commission E (the German expert committee established 1987 to evaluate the safety and efficacy of over 300 herbs and herb combinations sold in Germany).Uzara is used as a treatment for dysentery. No health hazards or side effects are known in conjunction with the proper administration of designated therapeutic dosages. **(Guenwald et al, 2000).** It is documented that the inhibitory action of Uzara on the motility of smooth muscle organs is through stimulation of the inhibitory sympathetic supply. This characteristic action is proved to affect the circular and longitudinal muscle of the whole intestinal tract, the urinary bladder and especially the pregnant uterus. After initial transient rise, the tone decreases with weaker and occasional propulsive action **(Blumenthal, 1998).** Traditional uses of Uzara include cramps (including afterbirth cramps), dysmenorrhea, and menstrual pain, said to be excellent for mild or painful menstruation. However, there are no studies addressing the use of Uzara in dysmenorrhea

**Aim of the study:**

To compare Uzara (*Xysmalobium undulatum*) and Ibuprofen (NSAID) in terms of efficacy, safety and tolerability in the treatment of moderate and severe dysmenorrhea.

**1. PROTOCOL OUTLINE**

**1.1 TITLE** A preliminary study of Uzara (Xysmalobium undulatum) and Ibuprofen in the treatment of primary dysmenorrhea among Egyptian Medical University Students: A randomized, crossover study

**1.2 STUDY SITE**

Ain Shams University Hospitals.

**2. STUDY OBJECTIVES**

**2.1 Primary Objectives**

2.1.1. To evaluate and compare the efficacy of Uzara (*Xysmalobium undulatum*) and ibuprofen 400 mg in the treatment of moderate and severe cases of primary dysmenorrhea.

**2.2 Secondary Objectives**

2.2.2. To evaluate and compare the side effects and tolerability of Uzara (*Xysmalobium undulatum*) and ibuprofen 400 mg in the treatment of moderate and severe cases of primary dysmenorrhea.

**3. STUDY DESIGN**

A pilot study to compare between both drugs; phase III prospective randomized, comparative two ways cross-over assignment, safety/efficacy study.

**3.1 POPULATION**

Sixty medical students having moderate or severe primary dysmenorrhea will be recruited to receive treatment and a written consent will then be obtained from each student before sharing in the comparative pilot study.

**3.2. STUDY TREATMENT AND METHODS**

**3.2.1. Interventional drugs:**

3.2.1.1. The study drug: Uzara 40 mg coated tablets,

• Containing purely active substance 40 mg dry extract of roots of *Xysmalobium undulatum* (L.) - uzara, (4-6:1) [extracting medium: methanol 60% (V/V)] and glucose, lactose, sucrose (sugar), and fortifying wheat (Uzara®).

• Registration owner: STADA GmbH, Stadastr. 2-18, 61118 Bad Vilbel, Germany. Manufacturer: HEMOPHARM GmbH, Koenigsteiner Strasse 2, 61350 Bad Homburg, Germany.

• Intake: Five days (maximum), orally 2 tablets/8hours for two doses then 1 tablet/8 hours. If there is mild or no pain 8 hours from the last dose, the use of drug should be stopped.

3.2.1. 2. Control drug: Brufen 400 mg sugar coated tablets,

• Each tablet containing 400 ibuprofen 2-(4 isobutylphenyl)-propionic acid, sugar coated, light magenta in color.

• Manufacturer: Kahira Pharm. & Chem. Ind. Co.(Cairo, Egypt) under license from Abbott Laboratories.

• Intake: Five days (maximum) orally 1 tablet/6hours. If there is mild or no pain 6 hours from the last dose, the use of drug should be stopped.

**3.3. STUDY ENTRY AND DURATION**

**3.3.1. Recruitment**

3.3.1.1. By the start, it will be announced during lectures that those students (medical and nursing students attending the hospital for medical training) suffering from dysmenorrhea and willing to volunteer in a trial of a new therapeutic line are invited to register themselves in a preliminary list for interview. Later on, they will be approached, interviewed (in order) with full explanation of the study protocol. Following provisional approval, assessment for eligibility will be performed. A questionnairewill be filled in **(CRF Ι).** This includes measurement of severity of dysmenorrhea, pain assessment (**CRF II- Baseline**), by pain scales; visual analogue scale (VAS), self‐reported and self‐rated verbal scale, and multidimensional scoring system (MDS).The presence of other associated symptoms [including headache, dizziness, mood changes, stomach cramps, nausea or vomiting, diarrhoea, constipation, edema, and weakness] will be subjectively scored (no, mild, moderate, and severe) and recorded.

3.3.1.2. Only those with moderate or severe dysmenorrhea will be included in the study after signing a written consent form (**CRF III**).

**Figure 1: Visual Analogue Scale (Ortiz et al, 2009)**


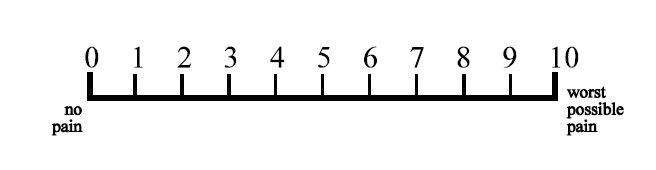


**Figure 2: Verbal Rating Scale (Daniels et al, 2008)**


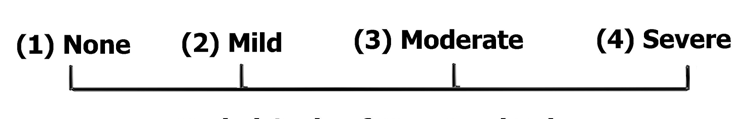


Table (1): multidimensional scoring system: (Ozerdogan et al, 2009)

| Severity grading | Working ability | Systemic symptoms | Analgesics |
| --- | --- | --- | --- |
| **Grade 0**  Menstruation is not painful and daily activity is unaffected  **Grade 1**  Mild. Menstruation is painful but seldom inhibits normal activity; analgesics are seldom required; mild pain  **Grade 2**  Moderate. Daily activity is affected; analgesics required and give sufficient relief so that absence from school is unusual; moderate pain  **Grade 3**  Severe. Activity clearly inhibited; poor effect of analgesics; vegetative symptoms (headache, fatigue, vomiting, and diarrhea); severe pain | Unaffected  Rarely affected  Moderately affected  Clearly inhibited | None  None  Few  Apparent | None required  Rarely required  Required  Poor effect |

**3.3.2. The Filling in the Questionnaire:**

3.3.2.1. All the participants will be assured that the information gathered through the survey will be kept confidential, being collected anonymously. The nature of the study will be explained and a signed written consent will be obtained. Exclusion and inclusion criteria will be applied before data collection. Pretreatment ultrasound screening will be subsequently performed to exclude any pelvic pathology.

**3.3.3. Sample Size Justification**

The current study is a pilot preliminary study. It is assumed that 60 students will be adequate to assess the efficacy and safety of the drugs used.

**3.3.3.1. Selection of participants:**

Using a computer-generated randomization system, the sixty included students, having moderate or severe dysmenorrhea, will be randomized to begin the treatment by one of both drugs then will receive the other drug the next cycle for purpose of cross-over assignment.

**3.3.3.2. Statistical analysis:**

Descriptive statistics for measured variables will be expressed as range, mean and standard deviation (for metric data); range, median and interquartile range (for discrete data); and number and proportions (for categorical data). Primary and secondary outcomes will be compared using Student t-test for continuous variables whereas the Pearson Chi square (χ2) test will be used for categorical variables. P<0.05 will be considered significant. Statistica 5.0 (StatSoft, Tulsa, OK, USA) will be used for data presentation and statistical analysis.

**3.3.4. Study Duration**

The duration of the study is 12 months.

**3.4. SELECTION OF PATIENTS**

**3.4.1. Inclusion Criteria**

1. Students of the Faculty of Medicine or High Institute of Nursing at Ain Shams University, having moderate or severe spasmodic dysmenorrhea (with the pain starting one day before or on the day of onset of bleeding and lasting at least a 1‐day during menstruation) requiring analgesic use for pain relief, with presence of moderate to severe dysmenorrhea in each of the last 3 cycles preceding admission into the study.
2. Age 18-28 years of age at the time of the study.
3. Regular cycles lasting 21 to 35 days with the actual menses periods lasting three to seven days.
4. Signed written informed consent by the student to participate in the study.

**3.4.2. Exclusion Criteria**

1. Students who are/were married, or planning to marry or take oral contraceptive pills during the study period.
2. Students on hormonal therapy during the last 6 months.
3. Known or suspected secondary dysmenorrhea (major abdominal or pelvic surgery, endometriosis, pelvic inflammatory disease (PID), ovarian cysts, pathological vaginal secretion, chronic abdominal pain, inflammatory bowel disease, irritable bowel syndrome)
4. History of significant chronic constipation &/or recurrent colitis.
5. Serious medical condition: Having the evidence of clinically relevant gynecological, cardiovascular, hematological, hepatic, gastrointestinal (especially active or severe peptic ulceration, or history thereof), renal, pulmonary (especially bronchial asthma or history thereof), endocrinology diseases, autoimmune diseases, neurologic or psychiatric disease, based on a clinical assessment and laboratory investigations.
6. Regular intake of medications that are not allowed in the study (pain medications for any other reason including NSAI drugs, digoxin, antidepressants, tranquilizers, hypnotics, sedatives, or sex hormones).
7. Any concomitant disease or condition that might require intake of analgesic medication.
8. Unwilling to comply with the protocol.
9. Participation in another clinical trial in the last 3 months prior to the start of this study

**3.5. RESULTS COLLECTION AND SCHEDULE**

**3.5.1. Enrollment (recruitment) Data [Case Record Form (CRF)]**

3.5.1.1. Following admission into the study, the case record form (CRF I) will be filled in including demographic information will be collected, including patient's age, parity, medical and surgical histories, and indications for the surgery.

3.5.1.2. Following admission into the study and pre-treatment, the subjects will be assigned to one out of two groups order of drug intake according to the computer-generated randomization plan. The interval between two successive menses will suffice as a wash out period. Each patient will have a Case Record Forms **(CRFII- A to F)** in which the results will be recorded for each cycle. The drug will be to be started once the onset of menstruation or pain is impending. The intended dosage is 2 tablets as a starting dose, then a tablet every 6-8 hours to complete the maximum of 5tab/day. The participants will record the pain intensity they experienced right before taking the medication (0 hour) and after 4, 12, 24, 48-60, 96-120 hours. For assessing the pain, visual analogue pain scale (VAS) will be used. Use of the other analgesics will be allowed for those suffering from a little or in case of no relief of pain. It will be permitted an hour or more after the intake of the test drug. On using this rescue or backup medication, the type, dose, frequency of administration and the response will be recorded. The presence of other associated symptoms will be subjectively scored (no, mild, moderate, and severe) and recorded. Data entailing possible adverse events to the drug will be collected. Tolerability will be assessed by recording adverse reactions during drug intake. At the termination of the 2 cycle study period (1 cycle for each drug), the participants will be asked to rank the preparations in order of effectiveness.

**3.5.2. Outcome measures:**

3.5.2.1. Primary outcome measures:

1. Pain intensity difference at each point (PID-point) and the sum of pain intensity difference at the six designated points (SPID-6).
2. Patient's global evaluation of study medication.
3. Difference in multidimensional scoring
4. The percentage of patients taking rescue medication
5. Time interval to rescue medication.

3.5.2.2. Secondary outcome measures:

1. Adverse events.
2. Drug tolerability.

**4. ETHICAL AND LEGAL ASPECTS**

**4.1. GOOD CLINICAL PRACTICE (GCP)**

The procedures set out in the study protocol, pertaining to the conduct, evaluation and documentation of this study, are designed to ensure that the investigators abide by the principles of good clinical practice.

**4.2. DELEGATION OF INVESTIGATOR RESPONSIBILITIES**

The investigator will ensure that all persons assisting with the trial are adequately informed about the protocol, any amendments to the protocol, the study treatments, and their trial-related duties and functions. The investigator will maintain a list of sub-investigators and other appropriately qualified person to whom he or she has delegated significant trial-related duties.

**4.3. PATIENT INFORMATION AND INFORMED CONSENT**

Before admission to the clinical study, the students must consent to participate after the nature, scope, and possible consequences of the clinical study have been explained in a form understandable to them. An informed consent document, in Arabic language, contains all locally required elements and specifies who informed the patient [CRF III]. After reading the informed consent document, the participant must sign the consent in writing. The participant’s consent must be confirmed at the time of consent by the personally dated signature of the participant and by the personally dated signature of the person conducting the informed consent discussions.

If the participant is unable to read, oral presentation and explanation of the written informed consent form and information to be supplied to participants must take place in the presence of an impartial witness. Consent must be confirmed at the time of consent verbally and by the personally dated signature of the participant or by a local legally recognized alternative (e.g., the patient’s thumbprint or mark). The witness and the person conducting the informed consent discussions must also sign and personally date the consent document. The original signed consent document will be retained by the investigator. The investigator will not undertake any measures specifically required only for the clinical study until valid consent has been obtained.

**4.4. CONFIDENTIALITY**

Only the participant number and participant initials will be recorded in the CRF, and if the participant’s name appears on any other document, it must be kept in privacy by the investigators. The investigator will maintain a personal participant identification list (patient numbers with the corresponding participant names) to enable records to be identified.

**4.5. PROTOCOL APPROVAL**

Before the beginning of the study and in accordance with the local regulation followed, the protocol and all corresponding documents will be declared for Ethical and Research approval by the Council of OB/GYN Department, Ain Shams University. Furthermore, the approval of the study protocol will be granted by Research Committee (REC), Faculty of Medicine, Ain Shams University (ASU), with presentation of patient’s information leaflet, consent form, and case‐record data form (CRF). The FMASU REC is organized and operated according to guidelines of the International Council on Harmonization (ICH) and the Islamic Organization for Medical Sciences (IOMS), the United States Office for Human Research Protections and the United States Code of Federal Regulations It operates under Federal Wide Assurance No. FWA00006444. Then, it will subsequently be registered in a recognized international registry.

**References**

**Banikarim C, Chacko MR and Kelder SH (2000): Prevalence and impact of**

**dysmenorrhoea on Hispanic female adolescents. Arch Paediatr Adolesc Med**

**154:1226-1229.**

**Biegelmayer C, Hofer G, Kainz C, Reinthaller A, Kopp B and Janisch H (1995): Concentrations of various arachidonic acid metabolites in menstrual fluid are associated with menstrual pain and are influenced by hormonal contraceptives.Gynecol Endocrinol 9:307-312.**

**Blumenthal M, ed. (1998): The Complete German Commission E Monographs: Therapeutic Guide to Herbal Medicines. Trans. S. Klein. Boston, MA: American Botanical Council.**

**Burke A, Smyth EM and FitzGerald GA (2006): Analgesic-Antipyretic and**

**Antiinflammatory Agents; Pharmacotherapy of Gout. In: Brunton L, Lazo J, Parker K, eds. Goodman & Gilman’s The Pharmacological Basis of Therapeutics, 11thed. New York: McGraw-Hill Professional: 671–716.**

**Daniels S, Gitton X, Zhou W, Stricker K and Barton S. (2008): Efficacy and tolerability of lumiracoxib 200 mg once daily for treatment of primary dysmenorrhea: results from two randomized controlled trials. J Womens Health (Larchmt); 17(3):423–37.**

**Davis AR and Westhoff CL. (2001): Primary dysmenorrhea in adolescent girls and treatment with oral contraceptives. J Pediatr Adolesc Gynecol; 14(1):3–8.**

**Dawood MY (2006): Primary dysmenorrhea: advances in pathogenesis and management. Obstet Gynecol 108:428- 441.**

**Dennehy CE (2006): The Use of Herbs and Dietary Supplements in Gynecology: An Evidence-Based ReviewJ MidwiferyWomens Health; 51:402–409 .**

**Durain D (2004): Primary dysmenorrhea: Assessment and management update. J. Midwifery Womens Health 49: 520 – 28.**

**El-Gilany AH, Badawi K and El-Fedawy S (2005): Epidemiology of dysmenorrhea among adolescent students in Mansoura, Egypt. Eastern Mediterranean Health Journal, 11(1/2): 155-163.**

**Fedele L, Parazzini F, Bianchi S, Arcaini L and Candiani GB (1990): Stage and localization of pelvic endometriosis and pain. Fertil Steril 53:155–158**

**Funk CD (2001): Prostaglandins and leukotrienes: Advances in eicosanoid biology. Science 294:1871-1875.**

**Guenwald J, Brendler T and Jaenicke C (2000): Physicians’ desk reference for herbal medicines, medical economics company.inc. at Montvale, NJ07645-1742,782.**

**Han SH, Hur MH, Buckle J, et al (2006): Effect of aromatherapy on symptoms of dysmenorrhea in college students: A randomized placebo-controlled clinical trial. J Altern Complement Med; 12:535–541.**

**Harel Z (2002): A contemporary approach to dysmenorrhea in adolescents.**

**Paediatr Drugs 4:797-805.**

**Harel Z (2006): Dysmenorrhea in adolescents and young adults: Etiology and management. J Pediatr Adolesc Gynecol 19:363-371.**

**Houston AM, Abraham A, Huang Z and D’Angelo LJ (2006): Knowledge, attitudes, and consequences of menstrual health in urban adolescent females. J Pediatr Adolesc Gynecol 19:271- 275.**

**Marchini M, Tozzi L, Bakshi R, Pistai R and Fedele L (1995): Comparative efficacy of diclofenac dispersible 50 mg and ibuprofen 400 mg in patients with primary dysmenorrhea. A randomized, double-blind, within-patient, placebo controlled study. Int J Clin Pharmacol Ther 33:491–497**

**Nigam S, Benedetto C, Zonca M, Leo-Rossberg I, Lubbert H and Hammerstein J (1991): Increased concentrations of eicosanoids and platelet-activating factor in menstrual blood from women with primary dysmenorrhoea. Eicosanoids 4:137-141.**

**Ortiz MI, Pérez-Hernández N, Macías A, Carrillo-Alarcón L, Rangel-Flores E, Fernández-Martínez E, et al. (2007): Drugs utilization for treating primary dysmenorrhea in university students. Rev Mex Cienc Farm; 38(4):24–9.**

**Ortiz MI, Rangel-Flores E, Carrillo-Alarco´n LC and Veras-Godoy HA. (2009): Prevalence and impact of primary dysmenorrhea among Mexican high school students. Int J Gynaecol Obstet; 107:240–3.**

**Ozgoli G, Goli M and Moattar F (2009): Comparison of effects of ginger, mefenamic acid, and ibuprofen on pain in women with primary dysmenorrhea the journal of alternative and complementary medicine volume 15, (2)129–132.**

**Ozerdogan N, Sayiner D, Ayranci U, Unsal A and Giray S. (2009): Prevalence and predictors of dysmenorrhea among students at a university in Turkey. Int J Gynaecol Obstet; 107:39–43.**

**Proctor ML and Murphy PA (2001): Herbal and dietary therapies for primary and secondary dysmenorrhoea. Cochrane Database Syst Rev**

**;( 3):CD002124.**

**Proctor ML, Murphy PA, Pattison HM, et al (2007): Behavioural interventions for primary and secondary dysmenorrhoea. Cochrane Database Syst Rev Jul 18; (3):CD002248.**

**Taylor D, Miaskowski C and Kohn J (2002): A randomized clinical trial of the effectiveness of an acupressure device (Relief Brief) for managing symptoms of dysmenorrhea. J Altern Complement Med; 8:357–370.**

**Case Record Form No. 1 [Enrollment Data]**

| **1** | **Serial Number** | |  | | | | | | | |  | | | | | | | |  | | | | | | |  |
| --- | --- | --- | --- | --- | --- | --- | --- | --- | --- | --- | --- | --- | --- | --- | --- | --- | --- | --- | --- | --- | --- | --- | --- | --- | --- | --- |
| **2** | **Name(Initials only)** | |  | | | | | | | |  | | | | | | | |  | | | | | | |  |
| **3** | **Study** | | (1) Medicine | | | | | | | | | | | | | | | | | (2) Nursing | | | | | | |
| **4** | **Past Medical History** | | (1) Irrelevant | | | | | | | | | (2) relevant | | | | | | | | Mention | | | | | | |
| **5** | **Past Surgical History** | | (1) Irrelevant | | | | | | | | | (2) relevant | | | | | | | | Mention | | | | | | |
| **6** | **Age (years)** | |  | | | | | | | | | | | | | | | | | | | | | | | |
| **7** | **Height (cm)** | |  | | | | | | | | | | | | | | | | | | | | | | | |
| **8** | **Weight (Kg)** | |  | | | | | | | | | | | | | | | | | | | | | | | |
| **9** | **Body Mass Index (BMI)** | |  | | | | | | | | | | | | | | | | | | | | | | | |
| **10** | **Religion** | | (1) Moslem | | | | | | | | | | | | | | | | | (2) Christian | | | | | | |
| **11** | **Family H. of dysmenorrhea:** | | (0) N/A | | | | | | | | (1) No | | | | | | | | 2) Mother | | | | | | | (3) Sister |
| **12** | **Age at Menarche (years)** | |  | | | | | | | | | | | | | | | | | | | | | | | |
| **13** | **Cycle duration (days)** | |  | | | | | | | | | | | | | | | | | | | | | | | |
| **14** | **Duration of menstrual flow (days)** | |  | | | | | | | | | | | | | | | | | | | | | | | |
| **15** | **Heaviness of flow (self-assessment):** | |  | | | | | | | | | | | | | | | | | | | | | | | |
| **16** | **Heaviness of flow** | | (no. of sanitary napkins used daily as menstrual at max. level) | | | | | | | | | | | | | | | | | | | | | | | |
| **17** | **Dysmenorrhea** | | (0) No | | | | | (1) Occasional | | | | | | | | | (2)Alternate Menses | | | | | | | | 4)Every Menses | |
| **18** | **Age at onset of dysmenorrhea (years)** | | | | | | | | | | | | (IF not applicable =99) | | | | | | | | | | | | | |
| **19** | **Duration of dysmenorrhea (days)** | | | | | | | | | | | |  | | | | | | | | | | | | | |
| **20** | **Onset of pain related to onset of menses (days):** | | (1) ‐2 | | | | | | | (2) ‐1 | | | | | (3) 0 | | | | | | (4) +1 | | | | | (5) +2 |
| **21** | **Maximal pain related to onset of menses (days):** | | (1) ‐2 | | | | | | | (2) ‐1 | | | | | (3) 0 | | | | | | (4) +1 | | | | | (5) +2 |
| **22** | **Maximal pain related to Flow of menses** | | (1) mild flow | | | | | | | | | | | (2) moderate | | | | | | | | (3) heavy | | | | |
| **23** | **Working ability during menses affected** | | (0) Never | | | (1) Rarely | | | | | | | | | | | (2) Moderately | | | | | | | (4) Clearly  Inhibited. | | |
| **24** | **Absence from work due to dysmenorrhea** | | (0) Never | | | (1)Occasional | | | | | | | | | | | (2)Alternate Menses. | | | | | | | (4) Every Menses. | | |
| **25** | **Medical consultation for dysmenorrhea** | | | (1) N/A | | | | | (2) No | | | | | | | (3)Yes direct | | | | | | | (4)Yes indirect | | | |
| **26** | **If yes, who**  **prescribed the drug** | (1)physician | | | | | (2)pharmacist | | | | | | | | | | | (3)relative | | | | | (4)other: mention | | | |
| **27** | **Use of analgesics** | (1) Never | | | | | | (2) rarely | | | | | | | | | | | (3) required | | | | | | | 4) highly required |
| **28** | **If YES, dose, rout of administration and frequency** | | | |  | | | | | | | | | | | | | | | | | | | | | |
| **29** | **Name of drug(s) (analgesics/antispasmodics) used if possible** | | | |  | | | | | | | | | | | | | | | | | | | | | |
| **30** | **Effect of analgesics** | | (1) N/A | | (2)marked  relief | | | | | | | | | | | | | | (3) moderate relief | | | | | | | (4) poor effect |

| **31** | **Associated symptoms during menses** | | | | |
| --- | --- | --- | --- | --- | --- |
|  | **Headache** | (1) No | (2) Mild | (3) Moderate | (4) Severe |
|  | **Dizziness** | (1) No | (2) Mild | (3) Moderate | (4) Severe |
|  | **Mood changes (nervousness)** | (1) No | (2) Mild | (3) Moderate | (4) Severe |
|  | **Stomach cramps** | (1) No | (2) Mild | (3) Moderate | (4) Severe |
|  | **Nausea/Vomiting** | (1) No | (2) Mild | (3) Moderate | (4) Severe |
|  | **Diarrhea** | (1) No | (2) Mild | (3) Moderate | (4) Severe |
|  | **Constipation** | (1) No | (2) Mild | (3) Moderate | (4) Severe |
|  | **Edema** | (1) No | (2) Mild | (3) Moderate | (4) Severe |
|  | **Weakness** | (1) No | (2) Mild | (3) Moderate | (4) Severe |

**Pain assessment: Baseline**

**Case Record Form II-A**

**Pain (A)**

- **Just Before Start of Medication: Point 1 Date: Time (24-format):**

|  | **Associated symptoms during menses** | | | | |
| --- | --- | --- | --- | --- | --- |
|  | **Headache** | (1) No | (2) Mild | (3) Moderate | (4) Severe |
|  | **Dizziness** | (1) No | (2) Mild | (3) Moderate | (4) Severe |
|  | **Mood changes (nervousness)** | (1) No | (2) Mild | (3) Moderate | (4) Severe |
|  | **Stomach cramps** | (1) No | (2) Mild | (3) Moderate | (4) Severe |
|  | **Nausea/Vomiting** | (1) No | (2) Mild | (3) Moderate | (4) Severe |
|  | **Diarrhea** | (1) No | (2) Mild | (3) Moderate | (4) Severe |
|  | **Constipation** | (1) No | (2) Mild | (3) Moderate | (4) Severe |
|  | **Edema** | (1) No | (2) Mild | (3) Moderate | (4) Severe |
|  | **Weakness** | (1) No | (2) Mild | (3) Moderate | (4) Severe |

**Absent From Wok due to dysmenorrhea** (1) No (2) Yes

**Pain assessment: Follow up**

**Case Record Form II-B (to II-F)**

**(4 /12/24/48-60/96-120) hours of Medication: (put check mark on the time point)**

|  | **Associated symptoms during treatment** | | | | |
| --- | --- | --- | --- | --- | --- |
|  | **Headache** | (1) No | (2) Mild | (3) Moderate | (4) Severe |
|  | **Dizziness** | (1) No | (2) Mild | (3) Moderate | (4) Severe |
|  | **Mood changes (nervousness)** | (1) No | (2) Mild | (3) Moderate | (4) Severe |
|  | **Stomach cramps** | (1) No | (2) Mild | (3) Moderate | (4) Severe |
|  | **Nausea/Vomiting** | (1) No | (2) Mild | (3) Moderate | (4) Severe |
|  | **Diarrhea** | (1) No | (2) Mild | (3) Moderate | (4) Severe |
|  | **Constipation** | (1) No | (2) Mild | (3) Moderate | (4) Severe |
|  | **Edema** | (1) No | (2) Mild | (3) Moderate | (4) Severe |
|  | **Weakness** | (1) No | (2) Mild | (3) Moderate | (4) Severe |

**Absent From Wok due to dysmenorrhea** (1) No (2) Yes

**Any other symptoms or changes on use of test drug: (1) No (2) Yes**

1. **If YES: Report:**
2. **Use of Any other analgesic (should be =>1 h after test drug): (1) No (2) Yes.**
3. **If YES:**
4. **􀂉 When? Date: Time (24-format):**
5. **􀂉 Name:**
6. **􀂉 Dose:**
7. **􀂉 Frequency:**
8. **􀂉 Response**

**CRF-III Informed Consent Form**

**لجنة أخلاقيات البحث العلمي - كلية الطب - جامعة عين شمس**

**عنوان البحث:**

**استخدام عقاري الأوتسارا والإيبوبرفين لعلاج عسر الطمث الأولي في الطالبات في كليات الطب المصرية.**

- **الخلفية العلمية و الھدف من إجراء البحث:**

**يُعد عسر الطمث من الاضطرابات النسائية الشائعة، فهو يؤثر في أكثر من نصف السيدات اللاتي يحضن، ويتراوح معدل انتشاره بين 50 و90% في العديد من الدراسات، وبالإضافة إلى كونه إضطراب نسائي إلا أنه يمثل مشكلة صحية هامة في الصحة العامة وطب الصناعات والممارسات العائلية حيث أنه يؤثر في نوعية الحياة والإقتصاد القومي نتيجة للتغيب قصير المدى من الجامعات وموقع العمل. وتُمثل مضادات الإلتهاب غير الإستيرودية بصورة واسعة خط العلاج الأول لهولاء السيدات، ولكن بعضهن لا يستجبن للعلاج، كما تٌوجد لهذا النوع من العقاقير موانع استخدام عديدة. إن الهدف من الدراسة هو المقارنة بين عقاري الأوتسارا والإيبوبرفين لعلاج عسر الطمث الأولي من ناحية الفاعلية والأمان وقابلية احتمال العقار.**

- **مكان البحث: مستشفيات جامعة عين شمس.**
- **عدد المشاركات بالبحث: 60 طالبة بكلية الطب و التمريض .**
- **أسلوب اختيار المشاركات في البحث: سوف يتم اختيار المشاركات تبعا لعوامل الاشتمال والاستبعاد.**
- **تفاصيل خطوات البحث:**

**ستقسم إلي مجموعتين: (المجموعة الأولى): تتكون من 30 طالبة سوف يستخدمن الايبوبروفين لمدة دورة واحدة ومع قياس درجة الالم وتحسن الاعراض المصاحبة لعسر الطمث والاعراض الجانبية للدواء.**

**أما (المجموعة الثانية): فتتكون من 30 طالبة يتم فيهن استخدام الأوتسارا ا لمدة دورة واحدة مع قياس درجة الالم وتحسن الاعراض المصاحبة لعسر الطمث والاعراض الجانبية للدواء.**

**ثم يتم تبادل العقار المستخدم بين المجموعتين لمدة دورة واحدة اخرى.**

- **الفوائد من البحث: في حالة ثبوت فاعلية وآمان وتحمل الأوتسارا سوف يمكن استخدامه كبديل لمضادات الإلتهاب غير الإستيرودية في علاج عسر الطمث الأولي وخاصة في حالة عدم إمكانية استخدامها لدواعي الآمان.**
- **المخاطر المحتمل حدوثھا: لقد تم استخدام عقاري البحث على مدى العديد من السنوات، وفي حالة استخدامها بصورة سليمة –كما هو في البحث الحالي- فإن لإحتمالية حدوث أية مضاعفات أو مخاطر للمشاركات في البحث بعيدة للغاية.**
- **التعويضات في حالة حدوث مخاطر من البحث: لا توجد تعويضات ولكن –وھو أمر روتيني- سوف يتم علاج أية مضاعفات بالمستشفى وليس على نفقة المريضة.**
- **البدائل المتاحة: في حالة رفضك المشاركة في ھذا البحث ستتلقين علاجك المعتاد.**
- **سرية المعلومات: سوف تعامل معلوماتك بسرية كاملة و لن يطلع على بياناتك سوى الباحث الرئيسي والمساعدين في البحث.**
- **حقك في الانسحاب: من حقك الانسحاب من البحث في أي وقت دون إبداء أية أسباب و دون أية عواقب سلبية عليك.**

**عند وجود أي استفسار لديك يمكنك الاتصال ب:**

**اسم الباحث الرئيسي:**

**أ.د./ كريم حسنين إسماعيل تليفون أرضي: 24140675تليفون محمول: 0123414212**

**اسم الباحث المشارك:رضوى منصور محمد زكى تليفون محمول: 0100037524**

**إقرار المريضة بالموافقة على الدراسة التالية:**

**"عسر الطمث الأولي بين طالبات الجامعة في مصر "**

**للإجابة على الجمل التالية: من فضلك ضعي دائرة حول نعم أو لا، مع العلم بأن ھذا يعني فھمك**

**لطبيعة الدراسة وأية مشكلات قد تطرأ نتيجة لذلك:**

**Patient's Random No.**

**Patient's Initials**

- **لقد قمت بقراءة دليل المعلومات المرفق نعم لا**
- **يمكنني الانسحاب من الدراسة في أي وقت نعم لا**
- **أعلم أن انسحابي من الدراسة - في حالة حدوثه- لن يؤثر سلباً على الرعاية الطبيةالمقدمة لى**

**نعم لا**

- **لقد حصلت على وقت كاف للسؤال عن أي شيء نعم لا**
- **أوافق على المشاركة في هذه الدراسة نعم لا**

**أقر أنني اطلعت و فھمت الإجراءات التي ستتم من خلال ھذا البحث و وافقت عليھا**

**المشارك في البحث**

**الاسم:**

**التوقيع:**

**التاريخ:**

**اسم الطالبة التوقيع التاريخ**

**اسم الطبيب التوقيع التاريخ**

*Ethical Committee of Scientific Research*

*Faculty of Medicine*  - *Ain Shams University*

CRF III: Consent from patients invited to participate in the research

***Research title: The use of Uzara (Xysmalobium undulatum) and Ibuprofen in the treatment of primary dysmenorrhea among Egyptian Medical University Students.***

**Introduction and Aim of the Work:**

Dysmenorrhea is one of the most frequent gynecologic disorders, affecting more than half of menstruating women. Prevalence of primary dysmenorrheal was reported in many studies to vary between 50 and 90 %. Besides being a gynecological problem, primary dysmenorrhea is an important health problem concerning public health, occupational health and family practice, as it affects both the quality of life and the national economy due to short-term school absenteeism and loss of labor. Unfortunately, both the prevalence of primary dysmenorrhea and the manner in which females attempt to solve this problem are unknown in most of the developing countries. Non-steroidal anti-inflammatory drugs (NSAIDs) are widely used as first-line therapy in women with primary dysmenorrhea. Some patients with primary dysmenorrhea do not respond to treatment with NSAIDs. In addition, some women have contraindications to these medications. The aim of this study is to compare Xysmalobium undulatum (L.) –Uzara and NSAIDs in terms of efficacy, safety and tolerability in treatment of dysmenorreah.

**Place of Work:**

The study will be carried out in Ain-Shams University Hospitals.

**Number of Participants:**

Sixty students studying medicine in Faculty of Medicine or High Institute for Nurses.

**Selection of Participants:**

Only those with moderate or severe dysmenorrhea and eligible for per participation as dictated by inclusion and exclusion criteria.

**Interventional drugs:**

the sixty students will be randomized into 2 groups, using a computer-generated randomization system then each group will begin the treatment by one of both drugs then will receive the other drug the next cycle for purpose of cross-over assignment. The participants will record the pain intensity they experienced right before taking the medication (0 hour) and after 4, 12, 24, 48-60, 96-120 hours. For assessing the pain, visual analogue pain scale (VAS) will be used. Use of rescue analgesic drugs, and the occurrence of side effects and tolerability will be recorded.

**Benefits Expected from the Study**:

If proved to be safe, effective and tolerable medication , uzara would be an alternative drug to NSAIDs and can be used in primary dysmenorrhea specially when NSAIDs are not suitable or contraindicated.

**Risk and Complications**

The used drugs have been prescribed over many years, and when properly used –as in the study- the occurrence of any complication or risk to the participant is highly remote.

**Compensation in case of complications:**

Both drugs are licensed to use, hence no health insurance is needed, yet in case of occurrence of any complication it will be managed (charge-free) –as routine- by the staff in Ain Shams University’s Hospitals.

**Alternatives to participating**

In case of refusing to participate in this research, the participant wishes will be respected and standard therapy will be advised.

**Confidentiality**

You will deal in complete confidentiality, and no one has right to read your patient medical information except the main researcher and his co-workers.

**Right to refuse or withdraw**

Any participant doesn't have to take part in this research if she doesn’t want. They may also stop participating at any time without declaring cause or suffering any negative consequences or penalty.

**Contact information**

Questions, concerns, or complaints: if you have any questions, concerns or complaints about this research study, its procedures, risks and benefits, or alternative courses of treatment, you should ask the investigator:

Prof. Dr. Karim Hassanin Ismail at Mobile number: 0123414212.

You can also call Dr. Radwa Mansour at mobile number: 0100037524, if there are any problems.

**Participants Consent Approving Volunteering in a Study**

***The use of Uzara (Xysmalobium undulatum) and Ibuprofen in the treatment of primary dysmenorrhea among Egyptian Medical University Students.***

**Kindly Encircle Yes or No, knowing that this signifies your understanding of the nature of the study and any realted subsubsequent problem**

**Patient's Random No.:**

**Patient's Initials:**

| - **I have read the accompanied explanatory leaflet** | **YES** | **NO** |
| --- | --- | --- |
| - **I can withdraw from the study any time** | **YES** | **NO** |
| - **I know that withdrawal from the study –in case of its occurrence- would not negative effects on health care provided to me.** | **YES** | **NO** |
| - **I have got enough time to ask about any issue.** | **YES** | **NO** |
| - **I agree to participate in this sudy** | **YES** | **NO** |

**Name of the Student: Signature:**

**Name of the Researcher: Signature:**

**Date:**
